# Treatment Pathways and Outcomes in Unresectable Gastric Cancer: A Retrospective Cohort Analysis

**DOI:** 10.7759/cureus.97146

**Published:** 2025-11-18

**Authors:** Yonatan Lessing, Tal Inbar-Weissman, Ron Cohen, Fahim Kanani, Adi Litmanovich, Adam Abu-Abeid, Boaz Sagie, Guy Lahat, Lior Orbach

**Affiliations:** 1 Division of Surgery, Tel Aviv Sourasky Medical Center, Tel Aviv, ISR; 2 Faculty of Medicine, Tel Aviv University, Tel Aviv, ISR; 3 Department of Transplantation, Rabin Medical Center, Petah Tikva, ISR; 4 Division of Surgery, Tel Aviv Sourasky Medical center, Tel Aviv, ISR

**Keywords:** advanced gastric cancer, cytoreductive surgery, gastrojejunostomy, hyperthermic intraperitoneal chemotherapy, palliative surgery, peritoneal metastasis, staging laparoscopy, survival outcomes

## Abstract

Background: The surgical management of advanced gastric cancer (AGC) ranges from diagnostic surgery (DS) to palliative procedures (PP) and cytoreductive surgery with hyperthermic intraperitoneal chemotherapy (CRS-HIPEC). This study aimed to describe real-world surgical strategies and evaluate their impact on patient outcomes.

Methods: We retrospectively analyzed 158 patients with AGC treated between 2014 and 2024 at a tertiary center and categorized them into three groups: DS (n=94), PP (n=52), and CRS-HIPEC (n=12). Demographic data, perioperative outcomes, and survival metrics were compared using ANOVA, Chi-square tests, and Kaplan-Meier estimates. In the CRS-HIPEC pathway, all 12 (100%) patients underwent staging laparoscopy, multidisciplinary team review, neoadjuvant chemotherapy, and interval CRS-HIPEC.

Results: Treatment selection was influenced by disease burden, symptom profile, and physiologic reserve. Patients undergoing CRS-HIPEC were younger (mean age 49.4 years, p=0.002) and had higher postoperative complication rates, nine of 12 (75.0%)-compared with PP, 21 of 52 (40.4%), and DS, seven of 94 (7.4%) (p<0.001). Median overall survival was longest after CRS-HIPEC (40.3 months), followed by PP (11.5 months) and DS (7.1 months). In the CRS-HIPEC cohort, median disease-free survival was 16.6 months.

Conclusions: Surgical strategies for unresectable AGC are heterogeneous and should be individualized. Palliative procedures can offer symptom relief and modest survival benefit in selected patients, though at the cost of morbidity and potential delays in systemic therapy. CRS-HIPEC, associated with prolonged survival in a small, highly selected cohort, should be regarded as hypothesis-generating. Multidisciplinary evaluation and careful patient selection remain central to optimizing outcomes.

## Introduction

Gastric cancer remains a leading cause of cancer-related mortality worldwide despite advances in diagnosis and multimodal therapy [1-3]. A substantial proportion of patients present with advanced gastric cancer (AGC) that is unsuitable for curative resection at diagnosis, or unresectability is revealed intraoperatively during exploration for putative curative intent [4-6]. In this setting, operative decision-making must balance symptom control and the possibility of oncologic benefit against the morbidity of intervention and the potential to delay or preclude systemic therapy, which remains the backbone of care for most patients with AGC [5,6].

Accurate appraisal of peritoneal dissemination is challenging. Even when cross-sectional imaging suggests resectability, staging laparoscopy frequently uncovers occult peritoneal disease. Prior series reports that up to approximately forty percent of patients without radiologic peritoneal metastases have macroscopic peritoneal carcinomatosis at laparoscopy, and an additional subset demonstrates positive peritoneal cytology in the absence of visible disease [7-10]. These findings have significant implications for the operative plan, perioperative risk, and subsequent systemic therapy.

Surgical strategies for AGC span three broad pathways: diagnostic surgery (DS), palliative procedure (PP), and cytoreductive surgery with hyperthermic intraperitoneal chemotherapy (CRS-HIPEC). DS, most commonly staging laparoscopy with or without biopsy, serves to confirm disease extent, establish non-curative status, and guide timely oncologic therapy with relatively low morbidity. PP (such as gastrojejunostomy, palliative gastrectomy, or surgical feeding access) is performed to alleviate or prevent complications like obstruction, bleeding, or refractory dysphagia. While PP can improve symptoms and occasionally extend survival, randomized and observational data highlight meaningful perioperative risk and uncertain oncologic advantage in unselected patients [11-13]. CRS-HIPEC has emerged as a potential option for carefully selected patients with limited peritoneal disease burden and favorable biology, typically after staging laparoscopy, multidisciplinary discussion, and neoadjuvant chemotherapy. However, prolonged survival has been reported in institutional series; generalizability remains limited, and selection/timing effects complicate interpretation [14-16].

The real-world comparative impact of these pathways is difficult to ascertain because patients triaged to PP or CRS-HIPEC often differ systematically from those undergoing DS, including tumor distribution (e.g., peritoneal carcinomatosis), symptom burden, and physiologic reserve. Moreover, the CRS-HIPEC pathway is inherently staged, with initial laparoscopy and cytoreduction candidacy assessment, multidisciplinary team (MDT) review, neoadjuvant chemotherapy, and interval CRS-HIPEC, which introduces lead-time and immortal-time biases that must be acknowledged when outcomes appear favorable.

In this single-center retrospective cohort study (2014-2024), we describe indications, perioperative outcomes, and survival across DS, PP, and CRS-HIPEC in patients with AGC deemed non-curative. We hypothesized that treatment selection reflects disease burden, symptom burden, and functional reserve. That pathway assignment would be associated with distinct perioperative profiles, likelihood of returning to systemic therapy, and overall survival. We aim to provide pragmatic data to inform MDT decision-making and to clarify where prospective, standardized selection criteria are most needed.

## Materials and methods

Study design and cohort

We performed a retrospective cohort study of consecutive patients with histologically proven gastric adenocarcinoma who underwent surgery at our center between 2014 and 2024. Patients were eligible if unresectability was established intraoperatively during exploration or if surgery was undertaken in a non-curative context for PP or CRS-HIPEC. Curative R0 gastrectomy and non-adenocarcinoma histology were excluded. For analysis, patients were grouped into three categories: DS, PP, or CRS-HIPEC. In the CRS-HIPEC pathway, all patients underwent staging laparoscopy, multidisciplinary team review, neoadjuvant chemotherapy, and interval CRS-HIPEC. The DS group included patients who did not undergo curative resection, either because they were scheduled for a planned staging laparoscopy from the outset or because they were taken to the operating room for planned curative gastrectomy based on preoperative imaging, but intraoperative findings of unresectable or metastatic disease led to abortion of curative resection and conversion to a diagnostic procedure only.

Variables and outcomes

Preoperative variables included age, sex, body mass index, comorbidities (hypertension, diabetes mellitus, ischemic heart disease, cerebrovascular disease, chronic renal failure, chronic obstructive pulmonary disease, and cognitive impairment), history of abdominal surgery, and receipt of neoadjuvant chemotherapy. Laboratory values recorded were albumin, hemoglobin, and carcinoembryonic antigen.

Operative variables included surgical approach, operative duration, estimated blood loss, and intraoperative mortality. Postoperative outcomes included complications classified by Clavien-Dindo (grade ≥3 considered significant), specific complications (delayed gastric emptying, anastomotic leak or intra-abdominal infection, postoperative bleeding, and respiratory complications), reoperation within 30 days, intensive care unit admission, length of stay, 30-, 60-, and 90-day mortality, readmission, and return to systemic chemotherapy. For oncologic adequacy where applicable, we recorded the extent of lymphadenectomy, total and metastatic lymph node counts, and margin status.

Statistical analysis and ethics

Statistical analyses were performed in SPSS software, version 29 (IBM Corp., Armonk, NY). Categorical variables were compared using Chi-square testing or the Freeman-Halton extension of Fisher’s exact test for 2×3 tables, as appropriate. One-way ANOVA compared continuous variables. Continuous variables are presented as means with standard deviations, and categorical variables as counts and percentages. Survival was estimated with Kaplan-Meier curves and compared using the log-rank test; medians are reported with 95% confidence intervals. Statistical significance was defined as p<0.05 (two-sided). The study was conducted in accordance with the Declaration of Helsinki and approved by the Tel Aviv Sourasky Medical Center Helsinki Committee (Institutional Review Board), approval TLV-0054-25 (22 January 2025), with a waiver of informed consent due to the retrospective design and use of de-identified data.

## Results

Perioperative data

Baseline demographics and preoperative characteristics differed across groups. While sex distribution did not reach statistical significance (p = 0.248), the proportion of females varied: DS 34 (36.2%), PP 17 (32.7%), and CRS-HIPEC 7 (58.3%). Patients undergoing CRS-HIPEC were significantly younger (mean 49.4 years) than those undergoing DS (63.9 years) or PP (65.7 years) (p = 0.002). Body mass index was comparable across groups (p = 0.883). Comorbidities were more prevalent in the PP cohort; chronic renal failure approached significance (PP seven (13.5%), p = 0.062). Neoadjuvant chemotherapy was universal prior to CRS-HIPEC (12 (100.0%)) and more frequent than in DS (44 (46.8%)) or PP (17 (32.7%)) (p < 0.001). Preoperative albumin did not differ significantly (p = 0.133). Mean preoperative hemoglobin was lower in PP and CRS-HIPEC versus DS (p = 0.029). Preoperative carcinoembryonic antigen did not differ significantly (p = 0.453). Baseline demographics and preoperative characteristics are summarized in Table 1.

**Table 1:** Baseline demographics and preoperative characteristics Values are presented as mean ± standard deviation or n (%). The p-values were calculated using one-way ANOVA for continuous variables and Chi-square or Fisher’s Exact test for categorical variables. DS = diagnostic surgery; PP = palliative procedure; CRS-HIPEC = cytoreductive surgery with hyperthermic intraperitoneal chemotherapy; BMI = body mass index; HTN = hypertension; COPD = chronic obstructive pulmonary disease; CEA = carcinoembryonic antigen; * = p < 0.05.

Characteristic	DS (n=94)	PP (n=52)	CRS-HIPEC (n=12)	Test statistic	p (two-sided)
Female sex	34 (36.2)	17 (32.7)	7 (58.3)	χ² = 2.80	0.248
Age, years	63.9 ± 14.2	65.7 ± 14.1	49.4 ± 15.5	F = 6.56	0.002 *
BMI, kg/m²	23.3 ± 6.7	23.9 ± 4.6	23.3 ± 4.5	F = 0.12	0.883
HTN	35 (37.2)	23 (44.2)	3 (25.0)	χ² = 1.71	0.426
Diabetes mellitus	15 (16.0)	10 (19.2)		χ² = 0.26	0.88
Ischemic heart disease	11 (11.7)	11 (21.2)	1 (8.3)	χ² = 2.84	0.245
Congestive heart failure	6 (6.4)	3 (5.8)	0 (0.0)	Fisher	0.999
Cerebrovascular disease	5 (5.3)	4 (7.7)	0 (0.0)	Fisher	0.865
Chronic renal failure	3 (3.2)	7 (13.5)	0 (0.0)	Fisher	0.062
COPD	4 (4.3)	1 (1.9)	0 (0.0)	Fisher	0.769
Past abdominal surgery	33 (35.1)	15 (28.8)	2 (16.7)	χ² = 1.95	0.377
Neoadjuvant chemotherapy	44 (46.8)	17 (32.7)	12 (100.0)	χ² = 29.74	<0.001 *
Albumin, g/L	37.9 ± 7.0	36.4 ± 5.3	40.4 ± 3.2	F = 2.08	0.133
Hemoglobin, g/dL	11.6 ± 1.6	10.8 ± 1.9	10.7 ± 0.9	F = 3.61	0.029 *
CEA, ng/m	10.4 ± 38.9	3.8 ± 5.6	3.7 ± 2.7	F = 0.80	0.453

Operative and postoperative course

Urgent surgery was uncommon (DS three (3.2%), PP four (7.7%), CRS-HIPEC 0 (0%); p = 0.458). There were no intraoperative deaths. Overall postoperative complications were higher after PP (21 (40.4%)) and CRS-HIPEC (nine (75.0%)) than after DS (seven (7.4%)) (p < 0.001). Severe complications (Clavien-Dindo ≥ 3) occurred in PP eight (15.4%) and CRS-HIPEC two (16.7%) versus DS three (3.2%) (p = 0.013). Delayed gastric emptying or feeding intolerance was most frequent after CRS-HIPEC three (25.0%) (p = 0.011). Anastomotic leak, fistula, or intra-abdominal infection occurred in PP 12 (23.1%) and CRS-HIPEC three (25.0%) versus DS three (3.2%) (p < 0.001). Intensive-care-unit admission was more common after CRS-HIPEC 7 (58.3%) and PP 12 (23.1%) than DS five (5.3%) (p < 0.001). Length of stay was longer after PP (21.8 ± 20.5 days) and CRS-HIPEC (18.6 ± 9.5 days) than DS (8.6 ± 9.6 days) (p < 0.001). Readmissions were more frequent after CRS-HIPEC five (41.7%) (p = 0.015). Thirty-, 60-, and 90-day mortality did not differ significantly. Return to systemic chemotherapy was higher after DS 74 (78.7%) than PP 17 (32.7%) or CRS-HIPEC four (33.3%) (p < 0.001). Operative details and postoperative outcomes are shown in Table 2.

**Table 2:** Operative details and postoperative outcomes Values are mean ± SD or n (%). Severe complications are defined as Clavien–Dindo grade ≥3. p-values calculated using one-way ANOVA for continuous variables and chi-square or Fisher’s Exact test for categorical variables. DS = diagnostic surgery; PP = palliative procedure; CRS-HIPEC = cytoreductive surgery with hyperthermic intraperitoneal chemotherapy; ICU = intensive care unit; DGE = delayed gastric emptying; LOS = length of stay; * = p < 0.05.

Outcome	DS (n=94)	PP (n=52)	CRS-HIPEC (n=12)	Test statistic	p (two-sided)
Urgent surgery	3 (3.2)	4 (7.7)	0 (0.0)	χ² = 1.56	0.458
Any postoperative complication	7 (7.4)	21 (40.4)	9 (75.0)	χ² = 44.18	<0.001 *
Clavien–Dindo ≥3	3 (3.2)	8 (15.4)	2 (16.7)	Fisher	0.013 *
DGE/feeding intolerance	3 (3.2)	1 (1.9)	3 (25.0)	Fisher	0.011 *
Leak/fistula/intra-abdominal	3 (3.2)	12 (23.1)	3 (25.0)	χ² = 20.21	<0.001 *
Bleeding — n (%)	1 (1.1)	1 (1.9)	1 (8.3)	Fisher	0.246
Respiratory complication	3 (3.2)	7 (13.5)	1 (8.3)	Fisher	0.052
Reoperation ≤30 days	2 (2.1)	5 (9.6)	1 (8.3)	Fisher	0.096
ICU admission	5 (5.3)	12 (23.1)	7 (58.3)	χ² = 28.92	<0.001 *
LOS, days	8.6 ± 9.6	21.8 ± 20.5	18.6 ± 9.5	F = 8.21	<0.001 *
Readmission	10 (10.8)	7 (13.5)	5 (41.7)	χ² = 8.42	0.015 *
30-day mortality	2 (2.2)	1 (1.9)	0 (0.0)	Fisher	0.999
60-day mortality	8 (8.8)	2 (3.8)	0 (0.0)	Fisher	0.429
90-day mortality	14 (15.4)	3 (5.8)	1 (8.3)	χ² = 3.12	0.21
Return to systemic chemotherapy	74 (78.7)	17 (32.7)	4 (33.3)	χ² = 34.55	<0.001 *

Surgery-specific characteristics

In the DS cohort (n = 94), 55 patients (58.5%) were taken to the operating room with curative gastrectomy as the planned procedure, but unresectability was discovered intraoperatively, and the operation was converted to a diagnostic procedure (laparoscopy and/or laparotomy with biopsy or limited palliation). Nineteen patients (20.2%) underwent planned staging laparoscopy, and 15 (16.0%) underwent exploratory laparotomy for disease assessment.

Diagnostic laparoscopy was performed in 61 (64.9%) cases, and unresectability was determined intraoperatively in 53 (56.4%). Laparotomy was performed in 43 (45.8%), and limited palliative procedures (gastrojejunostomy or feeding access) were performed in 16 (17.0%). Intraoperative findings included metastatic spread in 84 (89.4%), locally advanced disease in 27 (28.7%), and ascites in 36 (38.3%), with malignant cytology confirmed in 19 (52.8%) of those with ascites. Median overall survival in the DS cohort was 7.1 months (95% CI, 5.8-8.4).

In the PP group (n = 52), mean operative time was 3.5 ± 1.0 hours and median overall survival 11.5 months (95% CI, 5.6-17.3). Within the PP group (n = 52), procedures included gastrojejunostomy (n = 29, 55.8%), palliative gastrectomy (n = 15, 28.8%), and feeding jejunostomy or other feeding access (n = 8, 15.4%). Major complications (Clavien-Dindo ≥III) occurred in 26.7% of patients undergoing palliative gastrectomy, 13.8% after gastrojejunostomy, and none after feeding procedures. These patterns highlight the differing risk profiles of the various palliative approaches, with higher morbidity associated with more extensive resections.

Among patients in the CRS-HIPEC pathway, the median time from initial diagnostic or staging laparoscopy to the interval CRS-HIPEC procedure was 126 days (IQR 110-154). This interval reflects the multi-step process of multidisciplinary team evaluation and neoadjuvant chemotherapy and represents a period of guaranteed survival prior to CRS-HIPEC. In the CRS-HIPEC group (n = 12), mean operative time was 7.0 ± 1.3 hours; median disease-free survival was 16.6 months (95% CI, 1.3-30.7), and median overall survival was 40.3 months (95% CI, 0.0-86.8). Surgery-specific characteristics are presented in Table 3.

**Table 3:** Surgery-specific characteristics by cohort Values are n (%) or mean ± SD unless otherwise noted. DS = diagnostic surgery; PP = palliative procedure; CRS-HIPEC = cytoreductive surgery with hyperthermic intraperitoneal chemotherapy; GJ = gastrojejunostomy; SFJ = surgical feeding jejunostomy; OS = overall survival; DFS = disease-free survival; * = p < 0.05. Survival data expressed as median (95% CI). “—” indicates not applicable.

Outcome	DS (n=94)	PP (n=52)	CRS-HIPEC (n=12)	Test statistic	p (two-sided)
Urgent surgery	3 (3.2)	4 (7.7)	0 (0.0)	χ² = 1.56	0.458
Any postoperative complication	7 (7.4)	21 (40.4)	9 (75.0)	χ² = 44.18	<0.001 *
Clavien–Dindo ≥3	3 (3.2)	8 (15.4)	2 (16.7)	Fisher	0.013 *
DGE/feeding intolerance	3 (3.2)	1 (1.9)	3 (25.0)	Fisher	0.011 *
Leak/fistula/intra-abdominal	3 (3.2)	12 (23.1)	3 (25.0)	χ² = 20.21	<0.001 *
Bleeding	1 (1.1)	1 (1.9)	1 (8.3)	Fisher	0.246
Respiratory complication	3 (3.2)	7 (13.5)	1 (8.3)	Fisher	0.052
Reoperation ≤30 days	2 (2.1)	5 (9.6)	1 (8.3)	Fisher	0.096
ICU admission	5 (5.3)	12 (23.1)	7 (58.3)	χ² = 28.92	<0.001 *
LOS, days	8.6 ± 9.6	21.8 ± 20.5	18.6 ± 9.5	F = 8.21	<0.001 *
Readmission	10 (10.8)	7 (13.5)	5 (41.7)	χ² = 8.42	0.015 *
30-day mortality	2 (2.2)	1 (1.9)	0 (0.0)	Fisher	0.999
60-day mortality	8 (8.8)	2 (3.8)	0 (0.0)	Fisher	0.429
90-day mortality	14 (15.4)	3 (5.8)	1 (8.3)	χ² = 3.12	0.21
Return to systemic chemotherapy	74 (78.7)	17 (32.7)	4 (33.3)	χ² = 34.55	<0.001 *

Survival

**Figure 1:**
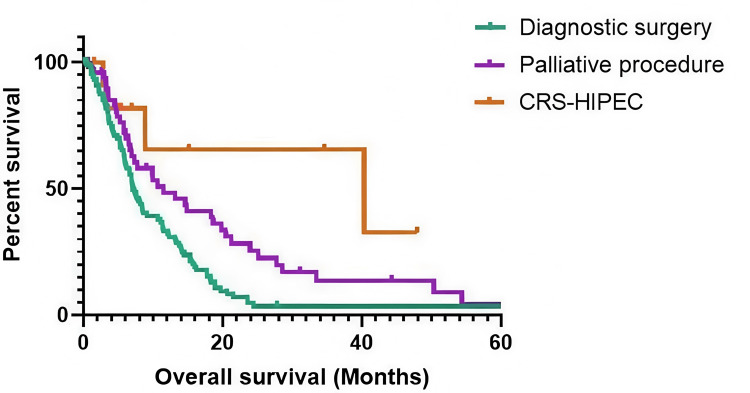
Kaplan-Meier survival curves comparing DS, PP and CRS-HIPEC for advanced gastric cancer Kaplan-Meier survival curves comparing overall survival among patients undergoing diagnostic surgery (DS), palliative procedures (PP), and CRS-HIPEC for advanced gastric cancer. This survival analysis demonstrates significantly improved median overall survival in patients undergoing CRS-HIPEC compared with those managed with diagnostic surgery or palliative procedures (p < 0.001, log-rank test). The Y-axis indicates overall survival probability; the X-axis indicates months after intervention.

Kaplan-Meier analysis demonstrated a clear separation of the overall survival (OS) curves by operative strategy. The DS curve showed the steepest early decline, consistent with advanced disease at presentation and limited therapeutic windows, whereas the PP curve was intermediate, reflecting symptom-driven intervention with higher perioperative risk and a lower likelihood of returning to chemotherapy. The CRS-HIPEC curve was most favorable, with prolonged tail behavior but wider confidence intervals due to the small, highly selected cohort. Median OS differed accordingly: CRS-HIPEC 40.3 months (95% CI, 0.0-86.8), PP 11.5 months (95% CI, 5.6-17.3), and DS 7.1 months (95% CI, 5.8-8.4), with a significant log-rank test for between-group differences. These patterns align with cohort characteristics (age, comorbidity, and neoadjuvant exposure) and the staged pathway required for CRS-HIPEC. The overall survival curve is shown in Figure 1.

**Figure 2:**
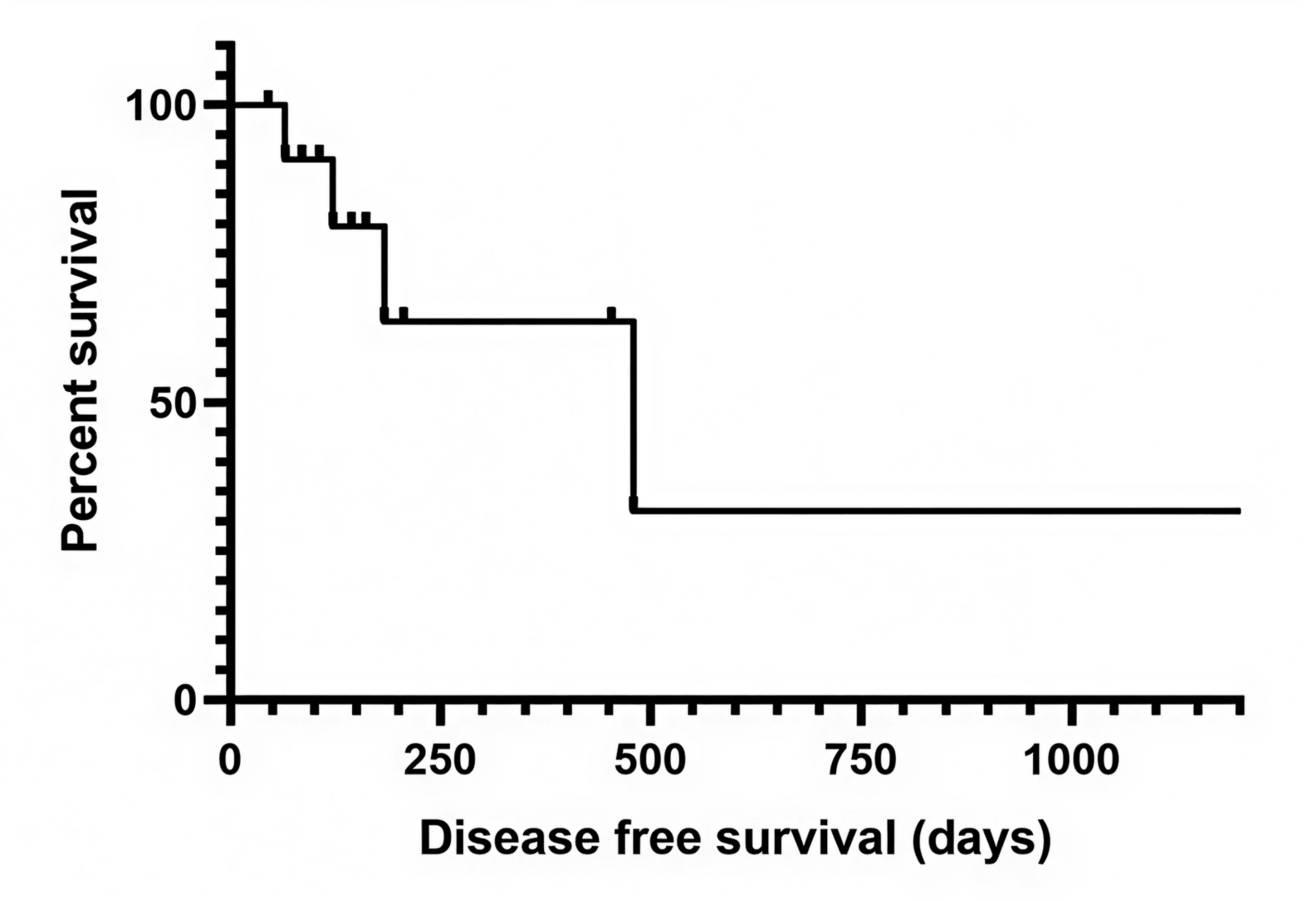
Kaplan–Meier curve showing disease-free survival after cytoreductive surgery with hyperthermic intraperitoneal chemotherapy (CRS-HIPEC) Kaplan–Meier analysis depicting disease-free survival following CRS-HIPEC in patients with advanced gastric cancer and peritoneal metastases. Median disease-free survival was approximately 420 days. Y-axis represents percent survival; X-axis represents disease-free survival in days.

Within the CRS-HIPEC cohort specifically, disease-free survival (DFS) was 16.6 months (95% CI, 1.3-30.7). The DFS curve shows early events and substantial right-censoring, which likely reflects both biological heterogeneity and the limited sample size; consequently, confidence bands widen over time, and estimates should be interpreted cautiously. Taken together with the OS findings, the DFS pattern underscores that even among carefully selected patients, recurrence remains common and durable disease control is achieved in a subset only, supporting our emphasis on patient selection, timing, and MDT-driven care pathways. The disease-free survival curve for the CRS-HIPEC cohort is shown in Figure 2.

## Discussion

This study provides a descriptive overview of real-world surgical pathways for patients with unresectable advanced gastric cancer, comparing perioperative outcomes and survival across three operative strategies: DS, PP, and CRS-HIPEC. These approaches represent different clinical intents-staging, palliation, and maximal cytoreduction and consequently attract patients with very different disease biology, symptom profiles, and treatment pathways. Understanding these distinctions is essential for individualized surgical decision-making in advanced disease.

Patients undergoing DS were typically explored with curative intent but found to be unresectable intraoperatively, most often because of occult peritoneal or locally advanced disease. Despite the absence of definitive resection, DS provided valuable staging information that guided subsequent therapy. This group demonstrated the lowest morbidity, the shortest hospital stays, and the highest rates of resuming systemic treatment, reflecting the benefit of early return to oncologic care. Nevertheless, survival remained poor (median 7.1 months), underscoring both the staging utility of laparoscopy and the dismal prognosis of radiologically undetected peritoneal spread [6,7]. These findings reaffirm the importance of DS as a diagnostic and therapeutic crossroad rather than as a futile procedure.

The PP cohort, in contrast, comprised patients operated on primarily for symptom relief-most commonly for gastric outlet obstruction, bleeding, or nutritional failure. These procedures, including gastrojejunostomy, palliative gastrectomy, and feeding access, were associated with higher morbidity, greater ICU utilization, and longer hospitalizations. Median survival modestly exceeded that of DS (11.5 vs. 7.1 months), yet only 17 (32.7%) patients resumed chemotherapy. Because continuation of systemic therapy remains the principal determinant of survival in AGC, the balance between symptom relief and the potential delay or loss of oncologic treatment is delicate [8-12,16-18]. PP should therefore be reserved for selected patients whose symptom burden justifies the operative risk and whose functional status allows postoperative recovery sufficient for systemic therapy.

The CRS-HIPEC group represented a small but distinct cohort, typically younger, with better performance status, limited peritoneal involvement, and universal receipt of neoadjuvant chemotherapy. These patients underwent the most extensive surgical intervention, combining cytoreduction with intraperitoneal chemotherapy to achieve maximal local control. Despite high operative complexity and substantial morbidity, this group achieved the most favorable oncologic outcomes, with a median overall survival of 40.3 months and disease-free survival of 16.6 months. These results mirror those of contemporary multicenter and registry studies, suggesting the potential benefit of CRS-HIPEC in biologically favorable cases [13-15,19]. However, the small sample size and inherent selection bias must temper the interpretation; it is likely that patient selection, rather than the procedure alone, accounts for much of the observed survival advantage.

**Figure 3:**
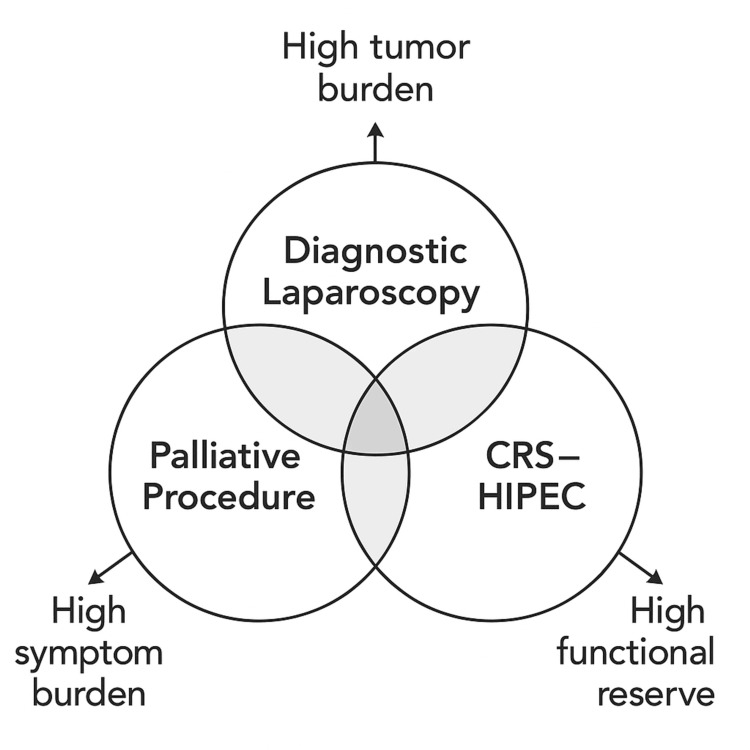
Conceptual overlap among surgical pathways in unresectable gastric cancer Venn diagram illustrating the conceptual gradients of tumor burden, symptom burden, and functional reserve that influence triage into one of three mutually exclusive surgical pathways: diagnostic surgery (DS), palliative procedures (PP), and CRS-HIPEC. The overlaps represent shared patient characteristics and decision-making considerations during multidisciplinary evaluation, rather than patients who underwent multiple procedural types.

Taken together, these findings highlight that no single operative strategy fits all patients with unresectable AGC. As shown conceptually in Figure 3, the three surgical pathways are mutually exclusive in this study, but they share overlapping domains of tumor burden, symptom burden, and functional reserve that guide multidisciplinary decision-making. DS remains the cornerstone of accurate staging and treatment planning. PP offers symptom control and a modest survival extension, but at the cost of higher morbidity and potential interruption of systemic therapy. CRS-HIPEC may achieve durable survival in exceptional cases characterized by limited peritoneal disease and a favorable response to chemotherapy. The overarching principle is to match surgical aggressiveness to oncologic reality, integrating biology, physiology, and patient goals within a precision-surgery framework [14,15,19].

This study has several limitations. Its retrospective, single-center design inherently introduces selection bias; patients were triaged to each surgical pathway based on disease burden, symptoms, and clinical judgment rather than standardized criteria. Yet this very selection process represents the essence of real-world surgical oncology: the nuanced, multidisciplinary decisions that weigh potential benefit against patient safety, quality of life, and limited therapeutic time. The heterogeneity of intent among the diagnostic, palliative, and cytoreductive groups reflects how care is individualized, not randomized, for patients facing an incurable disease.

While the small CRS-HIPEC cohort limits statistical power and generalizability, these data underscore the importance of thoughtful patient selection rather than procedural enthusiasm. For patients with unresectable gastric cancer, surgery often offers no cure, but it can still provide clarity, comfort, or meaningful time. Each operative decision must balance safety, expectations, and realism within a framework of compassion and multidisciplinary deliberation.

In particular, the CRS-HIPEC pathway introduces substantial immortal-time bias. To reach interval CRS-HIPEC, patients must survive and remain fit through multidisciplinary review and several months of systemic therapy. In our cohort, the median interval between diagnostic laparoscopy and CRS-HIPEC was 126 days, a period of guaranteed survival not experienced by patients in the DS or PP pathways. This lead-in period, combined with highly selective eligibility criteria, likely contributes to the longer overall survival observed in the CRS-HIPEC group and should temper direct comparisons of treatment effectiveness.

Future research should aim to refine patient selection through integration of radiologic, laparoscopic, and molecular markers that predict peritoneal disease biology. Prospective, multicenter studies are needed to determine whether CRS-HIPEC confers a true survival benefit beyond selection bias and to define standardized criteria for its use. Likewise, the role of PP in the modern systemic therapy era warrants reassessment, particularly in light of emerging intraperitoneal and immunotherapeutic strategies that may reshape the risk-benefit calculus of surgery in advanced disease.

## Conclusions

Surgical strategies for unresectable advanced gastric cancer are diverse and must be tailored to individual patients. Palliative procedures may provide symptom relief and modest survival benefit in carefully selected cases, though often at the cost of increased morbidity and potential delays in systemic therapy. CRS-HIPEC was associated with prolonged survival in a small, highly selected subgroup and should be interpreted as hypothesis-generating. Multidisciplinary evaluation, patient-centered discussion, and realistic goal setting remain central to optimizing care for this challenging population.
